# The immunopeptidomes of two transmissible cancers and their host have a common, dominant peptide motif

**DOI:** 10.1111/imm.13307

**Published:** 2021-02-04

**Authors:** Annalisa Gastaldello, Sri H. Ramarathinam, Alistair Bailey, Rachel Owen, Steven Turner, N. Kontouli, Tim Elliott, Paul Skipp, Anthony W. Purcell, Hannah V. Siddle

**Affiliations:** ^1^ School of Biological Sciences University of Southampton Southampton UK; ^2^ Department of Biochemistry and Molecular Biology and the Infection and Immunity Program Biomedicine Discovery Institute Monash University Clayton Victoria Australia; ^3^ Centre for Cancer Immunology University of Southampton Southampton UK; ^4^ Institute for Life Sciences University of Southampton Southampton UK

**Keywords:** contagious cancer, immunopeptidome, marsupial, MHC, Tasmanian devil, Transmissible cancer

## Abstract

Transmissible cancers are malignant cells that can spread between individuals of a population, akin to both a parasite and a mobile graft. The survival of the Tasmanian devil, the largest remaining marsupial carnivore, is threatened by the remarkable emergence of two independent lineages of transmissible cancer, devil facial tumour (DFT) 1 and devil facial tumour 2 (DFT2). To aid the development of a vaccine and to interrogate how histocompatibility barriers can be overcome, we analysed the peptides bound to major histocompatibility complex class I (MHC‐I) molecules from Tasmanian devil cells and representative cell lines of each transmissible cancer. Here, we show that DFT1 + IFN‐γ and DFT2 cell lines express a restricted repertoire of MHC‐I allotypes compared with fibroblast cells, potentially reducing the breadth of peptide presentation. Comparison of the peptidomes from DFT1 + IFNγ, DFT2 and host fibroblast cells demonstrates a dominant motif, despite differences in MHC‐I allotypes between the cell lines, with preference for a hydrophobic leucine residue at position 3 and position Ω of peptides. DFT1 and DFT2 both present peptides derived from neural proteins, which reflects a shared cellular origin that could be exploited for vaccine design. These results suggest that polymorphisms in MHC‐I molecules between tumours and host can be ‘hidden’ by a common peptide motif, providing the potential for permissive passage of infectious cells and demonstrating complexity in mammalian histocompatibility barriers.

## Graphical abstract

The Tasmanian devil has two independent transmissible cancers that circulate in the population. We have characterized the immunopeptidomes of both cancers and host cell line. We find that the cancers and host cell lines have a common peptide motif.

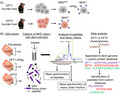

**Abbreviations**
DFT1: devil facial tumour 1
DFT2: devil facial tumour 2
ER: endoplasmic reticulum
IFN‐γ: interferon‐gamma
LC‐MS/MS: liquid chromatography–mass spectrometry/mass spectrometry
MHC: major histocompatibility complex
PBR: peptide binding region
PBS: phosphate‐buffered saline
RP‐HPLC: reversed‐phase high‐performance liquid chromatography
β_2_m: β_2_‐microglobulin

## Introduction

Transmissible cancers are clonal cell lines that can spread within a population through the passage of live cells between individuals. The emergence in nature of these types of cancers is rare, with only nine described so far, three in mammals and six independent transmissible cancers found in bivalve molluscs.^1^, ^2^ The mammalian transmissible cancers include the canine transmissible venereal tumour (CTVT) and two genetically independent transmissible cancers in the Tasmanian devil, devil facial tumour 1 (DFT1) and devil facial tumour 2 (DFT2).^3^, ^4^

Tasmanian devils are the largest surviving carnivorous marsupials found only on the Australian island of Tasmania, but since the emergence of DFT1,^5^ their number has declined significantly.^6^ The discovery in 2014 of a second independent transmissible cancer, termed DFT2, has the potential to dramatically increase the pressure on population survival.^3^, ^7^ Both cancers are thought to spread through biting behaviour, common in devils during feeding and mating, and cause death by starvation and/or organ failure within six to twelve months.^4^, ^8^

DFT1 and DFT2 are effectively allogeneic transplants and should be rejected by the host’s immune system through the engagement of classical major histocompatibility complex class I (MHC‐I) molecules on the cell surface of cancer cells with the T‐cell receptor (TCR) of CD8^+^ T cells. Classical MHC‐I molecules are found on all nucleated cells and are responsible for presenting intracellular peptides to CD8^+^ T cells, defining the immunological ‘self’ of an organism. Where pathogen‐derived peptides or cancer‐derived abnormal peptides are presented by MHC‐I, the cell may be recognized as infected or ‘non‐self’ and a cytotoxic response may be activated by T cells.^9^ Classical MHC‐I molecules are the most polymorphic genes in vertebrates, while non‐classical molecules possess more restricted polymorphism and expression, and having a range of functions.^10^

Mature cell surface MHC‐I molecules are a trimer, composed of an alpha chain, β_2_–microglobulin (β_2_m) and an antigenic peptide, usually 8–12 amino acids long, which binds in the peptide binding region (PBR) formed by the α1 and α2 domains of the alpha chain. The amino acids that define whether a peptide will bind to a MHC‐I molecule are termed anchor residues, and in most eutherian mammals are found at position two (p2) and the C‐terminus (pΩ) of the peptide sequence.^11^, ^12^ The binding specificity of an MHC‐I allele is determined by polymorphic amino acids that line the PBR and biochemically define a number of pockets that govern interaction with the anchor residues of the peptide ligand.^12^ This means that each MHC‐I allele has a preferred binding motif that generally describes the salient sequence features of the bound peptide ligands.

In devils, three polymorphic classical MHC‐I genes (Saha‐UA, Saha‐UB and Saha‐UC) and three non‐classical genes (Saha‐UD, Saha‐UK and Saha‐UM) have been identified; however, very little is known about their function or the nature of their ligands.^13^ Indeed, outside of well‐characterized species such as human and mouse and those of agricultural importance there has been little analysis of MHC peptide binding in wild species, with the exception of the bat.^14^, ^15^, ^16^

DFT1 cells express low levels of MHC‐I, explaining the lack of rejection by CD8^+^‐T cells.^17^ MHC‐I down‐regulation in these tumours is due to epigenetic mechanisms and can be reversed by treatment with the pro‐inflammatory cytokine interferon‐gamma (IFN‐γ).^17^ However, rare instances of spontaneous DFT1 tumour regression have been described and vaccination and immunotherapy of devils with live MHC‐I‐positive DFT1 cells demonstrate that devils can mount an effective immune response against the disease in some instances.^18^, ^19^, ^20^ We have recently shown that in contrast to DFT1, DFT2 cells express MHC‐I molecules, but the expressed alleles are similar between hosts and tumours in the few samples analysed.^21^

In recent years, the discovery of patient‐specific tumour neoantigens has enabled the development of robust experimental workflows for defining MHC‐I peptides in humans and mouse.^22^ Here, we have adapted and applied these techniques to define the repertoire of peptides (the immunopeptidome) presented by MHC‐I molecules in DFT1 + IFNγ, DFT2 and their host. Our data suggest that the peptide binding motif of MHC‐I molecules on DFT1 + IFNγ and DFT2 cells is similar to that of host cells, with a dominant peptide motif characterized by common p3 and pΩ residues. This is the first characterization of the immunopeptidome of a wild species and its transmissible cancers, providing the most detailed information to date on the antigenic landscape of transmissible cancers and a useful resource for the development of prophylactic peptide vaccines to protect the species in the wild.

## Materials and methods

**Figure 1:**
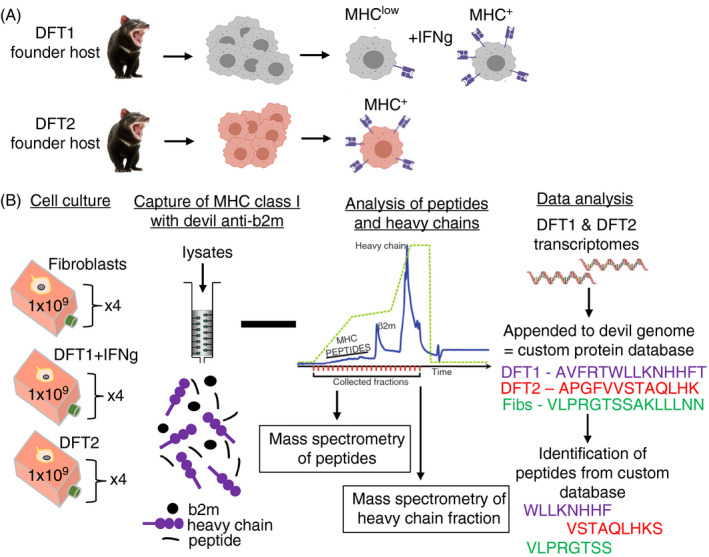
Overview of the experimental design to isolate and analyse MHC‐I‐bound peptides from two transmissible cancers (DFT1 and DFT2) and a healthy Tasmanian devil tissue. DFT1 and DFT2 arose independently in two founder devils (now dead) (A) and acquired transmissibility. DFT1 can be made MHC‐I‐positive in vitro with IFN‐γ, while DFT2 is MHC‐I‐positive in vitro without stimulation. (B) The experimental design to determine the MHC‐I allotypes on DFT1, DFT2 and fibroblast cells, along with the capture and analysis of MHC‐I‐bound peptides

The experimental setup utilized two transmissible cancer cell lines representing DFT1 and DFT2 (Figure 1A). The original founder devils for these cancers are no longer living, so for comparison a fibroblast cell line (used to sequence the Tasmanian devil genome) was used to represent a healthy host. The Tasmanian devil genome was appended to DFT1 and DFT2 transcriptomes to generate a custom protein database for identification of peptides from all three cell lines. An overview of the experimental design can be found in Figure 1B.

### Cell culture and IFN‐γ treatment

Three Tasmanian devil cell lines were analysed, the devil fibroblast cell line ‘Salem’ (referred to as fibroblasts in text), the DFT1‐derived cell line 4906 and the DFT2‐derived cell line RV (red velvet, referred to as DFT2), and are described in.^3^, ^21^, ^23^ To stimulate expression of MHC‐I molecules in DFT1, cells were cultured (17 h) in medium containing recombinant devil IFN‐γ described in,^17^ and are referred to as DFT1 + IFN‐γ in the text. To purify devil MHC‐I molecules by immunoaffinity purification, devil cells were expanded (1 × 10^9^ cells/replicate, *n* = 4) using Corning® HYPER*Flask®* M cell culture vessels (Sigma‐Aldrich), harvested and washed in 1X‐PBS before being snap‐frozen in liquid nitrogen and stored at −80°C. Media for cell growth and IFN‐γ treatment were batch‐prepared for consistency. Devil anti‐ β_2_m was used to assess total surface MHC‐I expression as previously described.^21^

### Whole‐cell proteomics

Whole‐cell proteomes were generated and analysed for DFT1 + IFN‐γ, DFT2 and fibroblast cells (*n* = 3) as described in Owen et al. 2020, submitted. The mass spectrometry proteomics data have been deposited to the ProteomeXchange Consortium via the PRIDE partner repository with the data set identifier PXD021784. RefSeq IDs from source proteins were searched on the NCBI RefSeq database to identify the Gene ID for analysis. Proteins that have not been previously annotated in the Tasmanian devil genome (https://www.ensembl.org/Sarcophilus_harrisii/Info/Index) were manually annotated based on protein homology to other species. Due to a lack of annotations for specific protein isoforms in the Tasmanian devil, multiple isoforms were discarded from functional analysis and a single Gene ID was used. Expression values for neural proteins were converted to a Log2 space, and pairwise comparisons were performed between DFT2 vs fibroblasts and DFT1 vs fibroblasts using a two‐sample Student’s *t*‐test (*P* values can be found in source data file 9, https://doi.org/10.5258/SOTON/D1585) to identify proteins with significantly differential expression between tumour and healthy cell lines. Source proteins significantly overexpressed in DFT1 + IFN‐γ or DFT2 compared with fibroblasts were visualized as a Volcano plot in RStudio using the geom_point function of the ggplot package.^24^

### DFT1 and DFT2 transcriptomes

Transcriptomes for the DFT1 and DFT2 cells were used to build custom databases for peptide identification (Figure 1). DFT2 cells (red velvet/DFT2_202) and DFT1 cells (4906) were cultured, and DFT1 cells were treated with IFN‐γ as described above. Cells were harvested, and mRNA was extracted using the Nucleospin RNA mini kit (Macherey and Nagel), following the manufacturer's instructions. The quality of RNA was determined using the Agilent 2100 Bioanalyzer. 1000 ng of RNA was used to generate strand‐specific RNA libraries. Libraries were sequenced on an Illumina HiSeq by Eurofins Genomics, generating over 64 million 150‐bp paired end reads for each cell line. Analysis is described in the ‘Data Analysis’ section below.

### Isolation of MHC‐I‐bound peptides by immunoaffinity purification

Peptides were isolated from four biological replicates of DFT1 + IFN‐γ, DFT2 and fibroblasts cells at ~1 × 10^9^ cells for each replicate, containing 10 pooled fractions per replicate. The high cell number was based on optimization experiments of immunoprecipitation of MHC‐I molecules by the anti‐devil β_2_m antibody and the amount of MHC‐I on the surface of the cells (as defined by flow cytometry above). However, once data were acquired for each cell line an additional experiment was performed on DFT2 cells only (*n* = 3) with only 1 × 10^8^ cells to validate the data. Peptides bound to MHC‐I molecules were isolated from cell lysates by immunoaffinity purification of devil β_2_m protein, purified by reversed‐phase high‐performance liquid chromatography (RP‐HPLC) and identified by liquid chromatography–mass spectrometry/mass spectrometry (LC‐MS/MS) as described in.^22^

Anti‐devil β_2_m antibody was purified by fast protein liquid chromatography (FPLC) from supernatant of hybridoma cells (identifier: 13‐34‐38). All chemicals were from Sigma‐Aldrich unless otherwise stated; all wash buffers and solutions were made using MS grade H_2_O and are detailed in.^22^

#### Generation of cell lysate

frozen cell pellets (1 × 10^9^ cells/replicate, *n* = 4) were ground using a cryogenic mill (Mixer Mill MM 400 (Retsch), 30 Hertz, 1 min × 2) followed by incubation (1 h, 4°C) with constant rotation in lysis buffer. Lysates were centrifuged (2000 × g, 10 min, 4°C) to remove nuclei, and supernatant was clarified by ultracentrifugation (100 000 × g, 45 min, 4°C).

#### Preparation of immunoaffinity columns

1·5 mL of protein A agarose resin (Repligen) was washed (PBS, 10 column volumes) before incubation with anti‐devil β_2_m antibody (10 mg in PBS) under constant rotation (4°C, 1 h). The antibody‐bound resin was washed with borate buffer, followed by freshly prepared triethanolamine to ensure there were no residual amines interfering with the cross‐linking reaction. The antibody was cross‐linked by incubating (1 h at RT) with dimethyl pimelimidate (40 mm in 0·2 m triethanolamine, pH 8·2), and the reaction was terminated by adding ice‐cold Tris. Unbound antibody was removed by washing with citrate buffer, and the column was washed with PBS until pH of flow through was >7.

#### Immunoaffinity purification of devil MHC‐I molecules

Cross‐linked columns were transferred to 4°C, and cell lysates were passed through the resin twice. Flow throughs were collected and stored at −80°C for subsequent proteomics analysis. The columns were washed at room temperature. MHC‐I molecules were eluted with 10% acetic acid.

#### Separation of MHC‐I eluate by RP‐HPLC

Eluted peptides were separated from MHC‐I alpha chains, β_2_m and other contaminants using a C18 reversed‐phase HPLC column (4·6 mm internal diameter × 10 cm, Chromolith Speed Rod, Merck) running on a mobile phase buffer of buffer A (0·1% trifluoroacetic acid (TFA)) and buffer B (80% acetonitrile/0·1% TFA) on an AKTAmicro^TM^ HPLC system (GE Healthcare) at the flow rate of 2 mL/min. The separation was performed with a rapid gradient of buffer A to B (2 to 40% B for 4 mins, 40 to 45% for 4 mins and a rapid 2‐min increase to 100% B) and 500 µl fractions collected on LoBind Eppendorf tubes. Fractions were concatenated in to 10 pools, vacuum‐dried (40°C), resuspended in 0·1% fresh formic acid (15 µl), sonicated (10 min) and stored at −80°C.

#### LC‐MS/MS analysis of peptides

Thawed samples were sonicated before addition of indexed retention time (iRT) peptide standards (1 pmole) and centrifugation (16 060 × g, 10 min) to remove any particulates. Peptides were trapped on a 2 cm Nanoviper PepMap 100 trap column at a flow rate of 15 μl/min using a RSLC nano‐HPLC. The trap column was then switched inline to an analytical PepMap 100 C18 nanocolumn (75 μm × 50 cm, 3 μm 100 Å pore size) at a flow rate of 300 nl/min using an initial gradient of 2·5% to 7·5% buffer B (0·1% formic acid 80% ACN) in buffer A (0·1% formic acid in water) over 1 min followed with a linear gradient from 7·5% to 32·5% buffer B for 58 min followed by a linear increase to 40% buffer B over 5 min and an additional increase up to 99% buffer B over 5 min. Separated peptides were analysed using a Q Exactive Plus mass spectrometer (Thermo Fisher Scientific, Bremen, Germany). Full‐scan MS spectra (*m*/*z* 375–1800) were acquired in the Orbitrap with 70 000 resolution (*m*/*z* 200) after the accumulation of ions to a 5 × 10^5^ target value with a maximum injection time of 120 ms. The 12 most intense multiply charged ions (z ≥ 2) were sequentially isolated and fragmented by higher energy collisional dissociation at 27% with an injection time of 120 ms, 35 000 resolution and target of 2 × 10^5^ counts. An isolation width of 1·8 *m*/*z* was applied, and underfill ratio was set to 1% and dynamic exclusion to 15 s.

An additional experiment was performed on DFT2 cells only (*n* = 3) with lower number of cells (1 × 10^8^). The MHC‐I molecules were isolated as described above, but peptides were separated by an Ultimate 3000 RSLC nano system (Thermo Scientific) using a PepMap C18 EASY‐Spray LC column, 2 µm pore size, 75 µm × 75 cm column (Thermo Scientific) in 0·1% formic acid and coupled online to an Orbitrap Fusion Tribrid Mass Spectrometer (Thermo Fisher Scientific, UK) with a nano‐electrospray ion source. Peptides were eluted with a linear gradient of 3%‐30% acetonitrile and 0·1% formic acid (v/v) at a flow rate of 300 nl/min over 110 min. Full scans were acquired in the Orbitrap analyser using the Top Speed data‐dependent mode, performing a MS scan every 3 s cycle, followed by higher energy collision‐induced dissociation MS/MS scans. MS spectra were acquired at resolution of 120 000 at 300 *m*/*z*, RF lens 60% and an automatic gain control ion target value of 4.0e5 for a maximum of 100 ms. MS/MS resolution was 30 000 at 100 *m*/*z*. Higher energy collisional dissociation fragmentation was induced at an energy setting of 28 for peptides with a charge state of 2–4, while singly charged peptides were fragmented at an energy setting of 32 at lower priority. Fragments were analysed in the Orbitrap at 30 000 resolution. Fragmented *m*/*z* values were dynamically excluded for 30 s.

#### Data analysis

Immunopeptidomics data were searched against a custom database for each cell line using PEAKS® X software (Bioinformatics Solutions Inc., Waterloo, ON, Canada), and peptide identities were determined by applying a false discovery rate cut‐off of 1%. A custom protein database was generated using RNAseq data from DFT2 and DFT1 + IFN‐γ cells to identify non‐synonymous SNP and indel variants, which were appended to the reference genome (https://www.ensembl.org/Sarcophilus_harrisii/Info/IndexDEVIL7.0). The DFT1 and DFT2 fastq files were aligned with the reference genome using HISAT‐2 (hierarchical indexing for spliced alignment of transcripts)^25^ and filtered for a mapping quality > 20 and a maximum read depth of 50 000 using SAMtools.^26^ Non‐synonymous SNP and indel variants were called using BCFtools^27^ and filtered for a minimum read depth of 10 and mapping quality of 20. R package customProDB was used as described in^28^ to generate protein sequences containing the identified variants. These sequences were then appended to the reference protein database to create the Sarcophilus_harrisii.DEVIL7.0.custom_db.fasta database that was used for proteomic searches.

**Figure 2:**
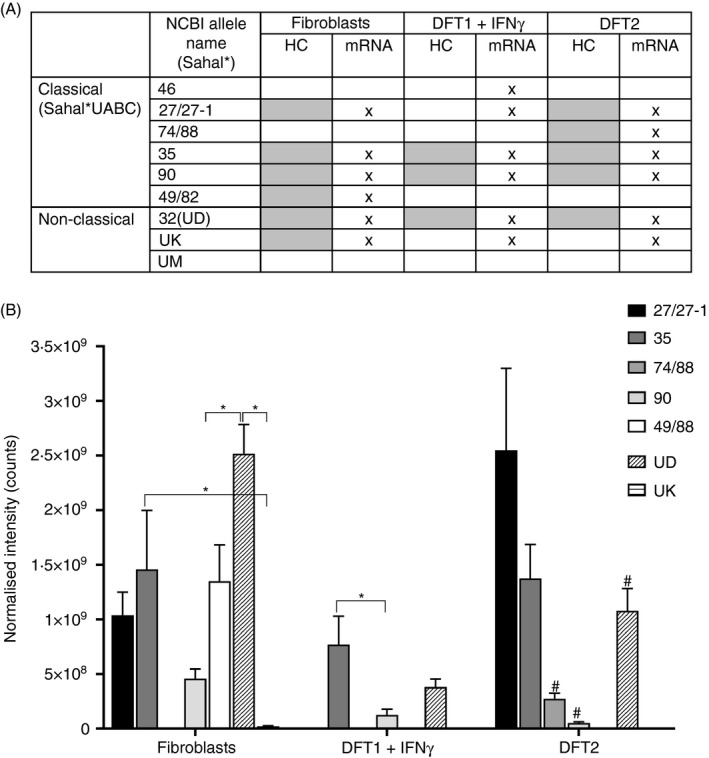
DFT1 + IFN‐γ and DFT2 cell lines have a restricted repertoire of MHC‐I alleles compared with devil fibroblasts. (A) Table of devil MHC‐I alleles identified in at least three out of four replicates in devil cell lines by analysis of the alpha chain fraction separated by HPLC in this paper (shaded boxes) or by mRNA analysis (crossed boxes) in a previous publication.^21^ A devil fibroblast cell line (referred to as fibroblasts) represents a host devil. DFT1 is represented by the cell line 4906 treated with interferon‐gamma to induce MHC‐I expression (DFT1 + IFN‐γ in the table and text), and DFT2 is represented by the cell line Red Velvet (DFT2 in the table and text). (B) Quantification of alpha chains of MHC‐I alleles in (A) represented as normalized intensity. Statistically significant comparison (*P* < 0·05) is shown with * & #; # = vs SahaI*27/27‐1 in DFT2, and raw data can be found in data file 1 (https://doi.org/10.5258/SOTON/D1585)

**Figure 3:**
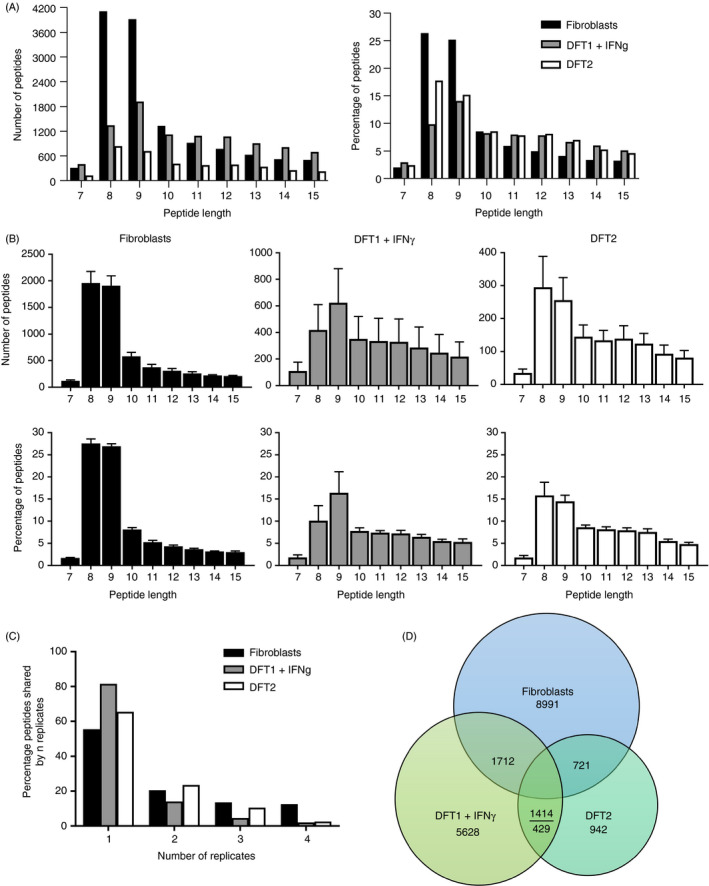
Peptide length distribution, reproducibility of peptidomics experiments and shared peptides between fibroblast, DFT1 + IFN‐γ and DFT2 cell lines. (A) Length distribution of unique peptide sequences across all replicates isolated from fibroblast, DFT1 + IFN‐γ and DFT2 cell lines. (B) Total number and percentage of peptides of different lengths isolated from devil cell lines. Data are mean ± SEM, *n* = 4/cell line. (C) The percentage of peptides found in 'n' replicates for each cell line. For full data set, see data file 2, https://doi.org/10.5258/SOTON/D1585. (D) Venn diagram showing unique 7–15mer peptides shared among fibroblasts, DFT1 + IFN‐γ and DFT2

**Figure 4:**
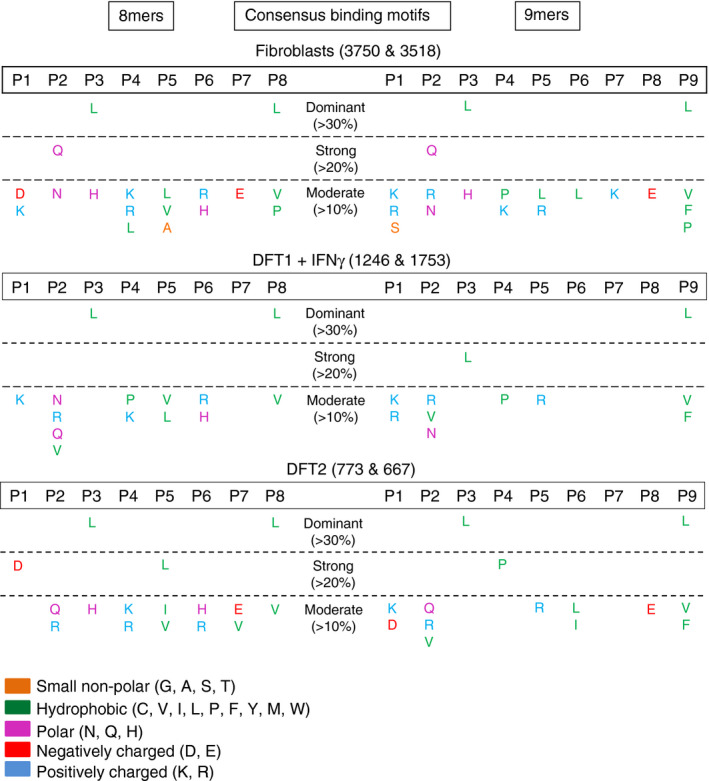
DFT1 + IFN‐γγ, DFT2 and fibroblasts share a preference for leucine anchor residues at p3 and pΩ. Tables of consensus binding motifs of 8mer and 9mer peptides in devil cell lines. Motifs were derived by calculating the frequency of each single amino acid at each position. Numbers after cell line names refer to the number of unique 8mers and 9mers analysed across replicates (*n* = 4). P1‐P9 = positions in the peptide sequence. Amino acids are represented by their single letter abbreviations and are coloured according to their biophysical properties based on the LESK scheme. For full data, see data file 4, https://doi.org/10.5258/SOTON/D1585

**Figure 5:**
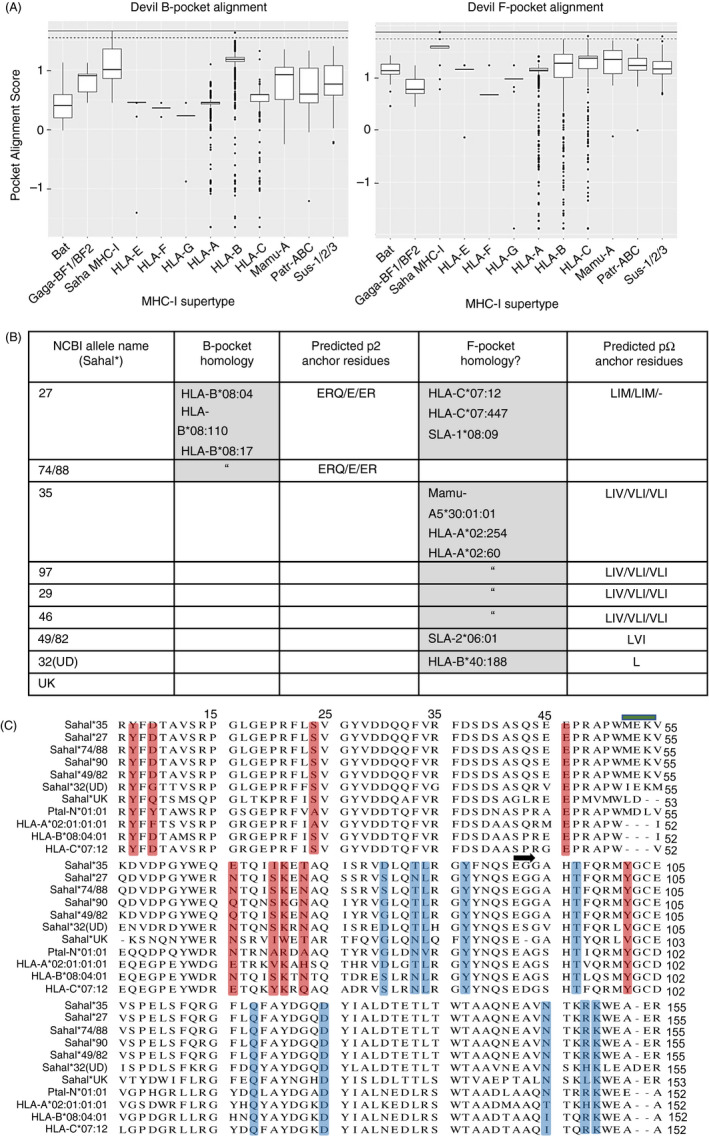
The F pocket of devil MHC‐I alleles is conserved with MHC‐I from eutherian mammals. (A) Binding pocket homology analysis of devil MHC‐I allele SahaI*27 against 16,748 MHC‐I sequences across 12 different MHC‐I supertypes from different species: Gaga = *Gallus gallus*, Mamu = *Macaca mulatta*, Patr = *Pan troglodytes, Sus = Sus scrofa*. Upper black line at the top of each box represents a perfect alignment score, data points above the tapered cut‐off line indicate sequence homology and were used to predict pocket motifs. (B) Table showing where homology was identified for B pocket and/or F pocket (grey boxes) for each devil MHC‐I allele; if predictions were possible, the respective motifs of the top 3 matches within that pocket have been included (p2/b pocket, pΩ/F pocket). The alleles with the highest homology to the devil are reported inside each grey box. (c) Multiple sequence alignment of α1‐α2 peptide binding groove region of devil MHC‐I alleles with selected human HLA alleles and the Ptal‐N*01:01 bat MHC‐I allele. HLA‐B*08 and HLA‐C*07 were included as they had the highest alignment scores to SahaI*27 with the B and F pocket, respectively. B pocket residues are highlighted in red, and F pocket residues are highlighted in blue. The blue bar denotes the 3aa insertion common to Ptal and devil MHC‐I sequences. A black arrow marks the beginning of the α2 domain

**Figure 6:**
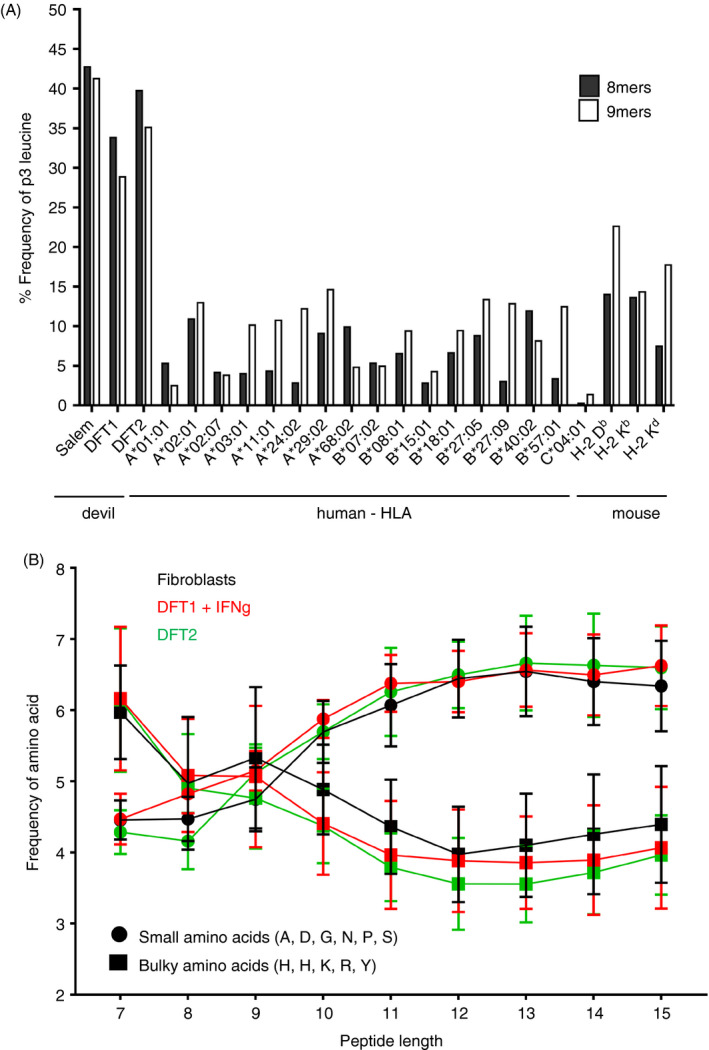
The strong preference for the amino acid leucine (L) at p3 in 8mers and 9mers is a unique characteristic of devil cell lines, while small versus bulky amino acid usage is similar to that of other species. (A) Observed frequency (%) of L at p3 in 8mer and 9mer peptides from devil cell lines (*n* = 4) compared with those calculated for selected mammalian alleles found in human and mouse. (B) Frequency of small and bulky amino acid usage was determined for each peptide length in the range of 7–15 amino acids. Alanine (A), aspartate (D), glycine (G), asparagine (N), proline (P) and serine (S) constituted small amino acids while phenylalanine (F), histidine (H), lysine (K), arginine (R) and tyrosine (Y) were considered bulky amino acids. Data are mean ± SEM. Data file 6, https://doi.org/10.5258/SOTON/D1585

**Figure 7:**
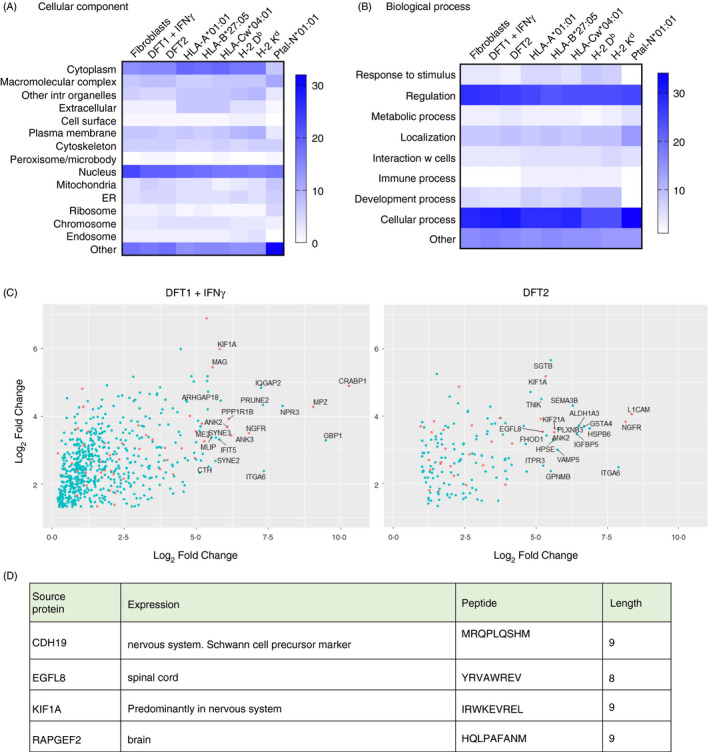
Neuronal proteins are a source of shared peptides in DFT1 + IFN‐γ and DFT2 cell lines. Heat maps representing the percentage of source proteins from different cellular components (A) or biological processes (B) for peptides isolated from fibroblasts, DFT1 + IFN‐γ and DFT2 compared with three MHC‐I alleles found in human being (HLA‐A*01:01, HLA‐B*27:05, HLA‐Cw*04:01), two found in mice (H‐2 D^b^, H‐2 K^d^) and one found in *Pteropus alecto* (the Australian black flying fox (Ptal‐N*01:01)). Higher percentages correspond to higher intensity of blue. (C) Volcano plots representing quantified proteins, which are a source of peptides and overexpressed in DFT1 + IFN‐γ (left) and DFT2 (right) compared with host fibroblasts. Proteins with expression restricted to the nervous system (neural proteins) are coloured in orange; proteins with the highest expression are labelled. (D) neural source proteins of 8mer and 9mer peptides found in both DFT1 + IFN‐γ and DFT2 cell lines but not in fibroblasts. For analysis of neural proteins, see data files 8 and 9, https://doi.org/10.5258/SOTON/D1585

The full peptidome dataset has been deposited to the ProteomeXchange Consortium via the PRIDE partner repository with the dataset identifier PXD020614. Analysis of peptides was performed using a combination of R 3.5.1 & RStudio 1.1.456 and Microsoft Office Excel. Some manual annotation of proteins was conducted where necessary. Consensus binding motifs were derived from the frequencies calculated for each amino acid at each position along the analysed peptides. Data files for all analyses used to generate Figures 2, 3, 4, 5, 6, 7 are openly available from the University of Southampton repository at https://doi.org/10.5258/SOTON/D1585.

Cellular localization and biological processes of source proteins were analysed using Software Tool for Researching Annotation of Proteins (STRAP^29^) by inputting UniProt devil IDs retrieved from Ensemble protein IDs. GO slim name analysis was performed using the Generic GO term Mapper (https://go.princeton.edu/cgi‐bin/GOTermMapper), to assign devil gene symbols to human GO slim terms. Ambiguous or unannotated gene symbols were automatically removed by the software. Results from both analyses were compared with six previously published human, mouse and bat MHC peptide data sets: annotations were compiled for three human MHC‐I alleles (HLA‐A*01:01, HLA‐B*27:05, and HLA‐Cw*04:01^30^, ^31^, ^32^), two murine alleles (H‐2D^b^ and H‐2 K^d^ ^33^) and one bat allele (Ptal‐N*01:01^16^), and analyses were performed as described above with the exception of murine data sets, which were mapped to murine gene symbols.

The frequency of small and bulky amino acid usage was calculated for each peptide length in the range 7‐15mers to capture as many potential MHC‐I peptides as possible. The frequencies of leucine at p3 in devil peptides were compared with those of other MHC‐I alleles in human being and mouse calculated from data available in the online database Immune Epitope Database and Analysis Resource (IEDB). Representative MHC‐I alleles were selected from available publications,^30^, ^31^, ^32^, ^33^, ^34^, ^35^, ^36^, ^37^ and only those binding both 8mers and 9mers were considered for comparison.

### Allotype identification of MHC‐I alpha chain fractions

The alpha chains of MHC‐I molecules isolated during immunoprecipitation were utilized for allotype identification using mass spectrometry. MHC‐I alpha chains from the immunoprecipitation (*n* = 4) were collected and adjusted to pH 8 followed by reduction with TCEP (12·5 mm, 60°C, 15 min) and alkylation (25 mm iodoacetamide, 15 min, RT). Proteins were then digested in‐solution using trypsin and chymotrypsin (16 h at 37°C and 25 °C, respectively). The resultant peptides were acidified, desalted using Bond Elut C18 solid‐phase extraction tips (Agilent) and subjected to mass spectrometry analysis as described above. Data obtained were searched against the Tasmanian devil protein database obtained from NCBI that included the MHC‐I sequences using MaxQuant software and the following settings: fixed modification: carbamidomethyl (C); variable modifications: acetyl (N‐term), oxidation (M); mass tolerance set to 20 ppm and false discovery rate set to 1%. Label‐free quantification data at peptide level were normalized to total peptide intensity in each sample prior to alpha chain quantitative analysis. The peptide allocation to each MHC‐I allotype was then manually checked by aligning peptides to the MHC‐I alleles defined in^38^ to ensure no duplicate assignments were made. Only variants found in at least three out of four replicates were considered for analysis of peptide intensities, and the raw data used to produce Figure 2B can be found in data file 1 (https://doi.org/10.5258/SOTON/D1585).

### Computational analysis of anchor pockets and epitope predictions

To investigate the B and F pockets of the devil MHC‐I in more detail, we used a combination of homology models and sequence alignment. This allowed us to predict the amino acids comprising the devil MHC‐I B and F pockets and make sequence comparisons across species, including species where more detailed peptide binding data are available. Pocket prediction for the devil MHC‐I alleles, including those found in the alpha chain fractions from fibroblasts, DFT1 + IFN‐γ and DFT2 cells, was performed using a custom R‐script available at: github.com/sct1g15/MHC_epitope_Prediction_Final. This script aligned 16,748 classical and non‐classical alleles across 7 different species to a devil MHC‐I query sequence using MAFFT multiple sequence alignment.^39^ Sequences used for the alignment were taken from the Immuno Polymorphism Database.^40^ Anchor pockets/epitopes of devil MHC‐I alleles were predicted by identifying sequence homology at the pocket level to MHC‐I alleles with experimentally defined peptide epitopes, allowing the inference of similar pocket motifs. This was achieved through implementation of the PMBEC point‐specific scoring matrix (PSSM), which was derived through competitive assays of combinatorial peptide mixtures to displace MHC‐bound and MHC‐tagged peptide.^41^ This approach assumes that amino acid modifications to the peptide ligand used to calculate the PSSM would have similar impact on binding affinity when imposed on the receptor sequence. Query sequences were scored through sequence similarity using the PSSM, at the specified pocket positions, which are predefined (residues defined below). A cut‐off point of 0·93*Maximum alignment score was used to determine relevant hits. Each residue contribution to the final alignment score was weighted by its probability to contact the PBR defined through the COACH binding site prediction server following I‐TASSER homology modelling of the query sequence.^42^ This allowed inclusion of adjacent residues enabling appropriate scoring with minor perturbations in pocket sites between MHC‐I alleles and incorporation of static structural information. Two predefined pocket sites were included: B pocket composed of residues 7, 9, 24, 34, 45, 63, 66, 67, 70 and 99, and F pocket composed of residues 77, 80, 81, 84, 94, 115, 122, 142, 145, 146 and residues adjacent to those defined. All positions are set relative to a HLA‐A2:01:01:01 reference sequence after multiple sequence alignment. Pocket epitopes were predicted using NetMHCpan 4.0^43^ binding affinity motifs for high alignment hits. This method prevents unsuitable prediction in the absence of representative training data for novel pockets present in devil alleles. Bat, chicken and devil MHC‐I sequences included in the alignment set were not used for epitope prediction.

## Results

### DFT1 + IFN‐γ and DFT2 cells have a restricted repertoire of MHC‐I alleles compared with fibroblasts

Analysis of the MHC‐I allotypes on each cell line showed that DFT1 + IFN‐γ and DFT2 cells have a more restricted repertoire compared with fibroblasts, with the majority of allotypes shared between the cell lines (shaded boxes in Figure 2A). The fibroblast cell line has the greatest diversity of cell surface MHC‐I molecules with six allotypes in total (SahaI*27/27‐1, SahaI*35, SahaI*90, SahaI*49/82, SahaI*32(UD) and SahaI*UK); DFT1 + IFN‐γ cells have the lowest diversity with three (SahaI*35, SahaI*90 and SahaI*32(UD), while DFT2 cells have five variants (SahaI*27/27‐1, SahaI*74/88, SahaI*35, SahaI*90 and SahaI*32(UD)). When DFT1 + IFN‐γ and DFT2 are considered together, there is only one unique allotype compared with fibroblasts, SahaI*74/88 found in DFT2 cells. DFT1 + IFN‐γ cells have no unique allotypes when compared to fibroblasts. SahaI*27 and SahaI*27‐1 were grouped together as the latter differs from SahaI*27 by one non‐synonymous substitution in the α2 domain of the alpha chain and could not be distinguished based on the peptides analysed.

Analysis of the relative abundance of each MHC‐I allotype showed that SahaI*27/27‐1, SahaI*35 and the non‐classical SahaI*32(UD) are the most consistently expressed across the cell lines. MHC‐I SahaI*32(UD) was the most abundant alpha chain in fibroblast lysates (Figure 2B), while SahaI*27/27‐1 was the most abundant in DFT2 and SahaI*35 and SahaI*32(UD) were the most abundant variants in DFT1. The only allotype found in DFT2 and not in fibroblasts, SahaI*74/88, had a relatively low abundance. Saha*UK was only present in fibroblasts and was the least abundant MHC‐I allotype.

### Tasmanian devil MHC‐I molecules preferentially bind peptides of 8 and 9 amino acids in length

Immunoaffinity purification with a devil‐specific anti‐ β_2_m antibody was used to isolate the peptides from DFT1 + IFN‐γ, DFT2 and fibroblast cell lines (Figure 1B). In total, from all three cell lines (*n* = 4), we isolated 25 554 unique peptides that were 7–15 amino acids, the expected length preference for MHC‐I molecules (7–15mers; table 1). Analysis of the length distribution of unique peptides (Figure 3A,B) revealed that 8mers were more abundant than 9mers in fibroblasts and DFT2 cells but not in DFT1 + IFN‐γ cells where 9mers were most abundant. Shorter and longer peptides were identified with lower frequency, while similar proportions of 10mers were found in all cell lines (Figure 3B).

**Table 1:** Number of total and unique peptide sequences in a devil fibroblast cell line and in devil facial tumour 1 and 2 (DFT1 + IFN‐γ and DFT2) cell lines

Cell line	No. of peptides identified (*n* = 4)	Unique sequences	Unique sequences 7–15mers (% of total)
fibroblasts	28 198	15 581	12 838 (82)
DFT1 + IFN‐g	17 280	13 636	9207 (67)
DFT2	6879	4648	3509 (75)
Total	52 357	33 865	25 554 (75)

Peptides were isolated by immunoaffinity purification with a pan‐MHC‐I antibody against devil β_2_m followed by separation with HPLC and analysis by mass spectrometry (*n* = 4/cell line). The fibroblast cell line (fibroblasts) yielded the highest number of unique 7–15mer peptide sequences (12 838) across the 4 replicates, with lower numbers isolated from DFT1 + IFN‐γ (9207) and DFT2 (3509). The number of peptides identified correlates with cell surface β_2_m expression on each cell line, with fibroblasts showing the highest expression, DFT2 the lowest and DFT1 + IFN‐γ the intermediate.

We calculated the percentage of 7–15mers found in either one, two, three or four replicates across all three cell lines. This analysis showed that the greatest proportion of peptides was found in only one replicate for all three cell lines, with DFT1 + IFN‐γ displaying the highest single replicate bias (Figure 3C). DFT1 + IFN‐γ was also the cell line with the lowest level of surface β_2_m, as assessed by flow cytometry (Figure S1). The three cell lines shared 1414 unique 7–15mers (Figure 3D), while DFT1 + IFNγ /fibroblasts, DFT2/fibroblasts and DFT1 + IFNγ /DFT2 shared, respectively 1712, 721 and 429 unique sequences 7‐15 amino acids in length.

### Peptides from fibroblasts, DFT1 + IFN‐γ and DFT2 possess similar anchor residues found at p3 and pΩ sites

The consensus binding motifs of peptides isolated from each cell line show striking similarities, despite the differences in MHC‐I allotype expression between cell lines. The amino acid frequencies were calculated for each amino acid at each position in 8mers and 9mers and classified as dominant (>30%), strong (20‐30%) and moderate (10‐20%) (Figure 4).

The 8mer and 9mer peptides had a preference for the hydrophobic amino acids leucine (L), valine (V), phenylalanine (F) or proline (P) at the C‐terminus in all three cell lines (Figure 4), a characteristic common to many eutherian MHC‐I presented peptides. All three cell lines also have a preference for L at p3 in both 8mers and 9mers, with this being dominant in fibroblasts and DFT2 and strong in DFT1 + IFN‐γ. Alongside this dominant motif, there are also features specific to each cell line. Glutamine (Q) was strong at p2 in 8mers and 9mers from fibroblasts, while aspartic acid (D) was strong at p1 and L at p5 for DFT2 8mers and P was strong at p4 in 9mers from DFT2.

To further validate the consensus motif, additional immunopeptidome experiments were performed on a smaller number of DFT2 cells (1 × 10^8^/replicate). 4450 peptides were isolated (*n* = 3), and the distribution of both the number and percentage of total 7mers to 15mers (Figure S2a) was similar to that of peptides from previous experiments using 1 × 10^9^ cells/replicate of DFT2 cells (Figure 3). Consensus binding motifs (Figure S2b) supported the finding of a dominant motif with hydrophobic anchors at pΩ (L or F) and p3 (L) (Figure 4) and potential additional anchors at p1 and p5 in 8mers, and at p4 in 9mers.

### Devil MHC class I F pocket, but not B pocket, shows homology with MHC class I molecules from eutherian mammals

In the absence of structural data for devil MHC‐I molecules, homology modelling and intraspecies pairwise sequence comparisons reveal differences in the classical binding pockets of the devil MHC‐I that are consistent with p3 and pΩ anchors. Ten devil MHC‐I alleles were modelled against the recently described bat (*Pteropus Alecto*) Ptal‐N*01:01 structure^14^ to predict both the B pocket (accommodating p2) and F pocket (accommodating pΩ) conformations. The bat Ptal‐N*01:01 allele was used for the analysis as it shares a 3aa insertion in the α1 domain with devil MHC‐I when compared to other eutherian MHC‐I alleles and therefore could provide a more matched homology model. Conformation‐weighted pairwise comparisons were made against 16,748 MHC‐I sequences from seven species (predominantly human) (Figure 5A). Only two devil MHC‐I alleles showed a high degree of conservation in their sequence across the predicted B pocket: SahaI*27 and SahaI*74/88 (Figure 5A left panel and 5b). Both alleles were identical to HLA‐B*08:04 at the residues making up the B pocket resulting in the same predictions (Figure 5C). Across the F pocket (Figure 5A, right panel), seven devil MHC‐I alleles showed high conservation in their sequence, four of which were identical predictions due to identical F pocket sequences (Figure 5B). Only SahaI*90 and SahaI*UK alleles did not have a predicted F pocket. Predicted anchor residues at the B pockets of SahaI*27 and SahaI*74/88, but an absence of homology at the B pocket for the remaining alleles, are consistent with the experimentally derived consensus binding motifs that predict only a secondary anchor at p1 or p2. Further, the high similarity of the F pocket supports the presence of a hydrophobic pΩ anchor.

### Dominance of leucine (L) at p3 of 8mers and 9mers is a unique characteristic of devil peptides

Leucine is prevalent at pΩ in many human and murine MHC‐I alleles, while dominance of L at p3 is less common. We compared the frequency of L at p3 in devil peptides to a range of well‐characterized MHC‐I allotypes found in human being and mouse (Figure 6A). Leucine was much more frequent at p3 in 8mers and 9mers isolated from devil cell lines than among peptides recognized by a variety of human and murine alleles. The alleles with the highest frequency of L at p3 among 9mers were the murine H‐2 Db and H‐2 Kd, although they did not reach frequencies found in devil. The percentages of small and bulky amino acids present in devil MHC‐I‐bound peptides were also calculated (Figure 6B). As reported previously for other species,^16^, ^30^, ^32^, ^33^ the use of small amino acids increased, whereas the frequency of bulky amino acids decreased with increasing peptide length in devil.

### Peptides derived from neural‐specific source proteins are presented on MHC‐I molecules in DFT1 + IFN‐γ and DFT2

Annotations for source proteins of peptides in fibroblasts, DFT1 + IFN‐γ and DFT2 were retrieved and compared with those from three human MHC‐I alleles, two mouse alleles and one bat allele (Figure 7). The greatest percentage of peptides were derived from either nuclear or cytoplasmic proteins (Figure 7A) and involved in regulation of cellular processes (Figure 7B), for all analysed cells/alleles.

As DFT1 + IFN‐γ and DFT2 are predicted to have the same cellular origin, a Schwann cell,^44^, ^45^, ^46^ we identified the peptides that derive from source proteins with a neural function and are specific to or overexpressed in DFT1 + IFN‐γ and/or DFT2 compared with fibroblasts (Figure 7C). In DFT1 + IFN‐γ, 54 peptides (8mers and 9mers) were derived from 45 neural proteins, which were not a source of peptides in fibroblasts. In DFT2, 15 peptides (8mers and 9mers) were identified that derived from 17 neural source proteins and did not appear in the fibroblast immunopeptidome (Figure 7C). Four of these source proteins were shared between DFT1 + IFNγ and DFT2 and generated one shared 8mer (source protein: EGFL8) and 3 shared 9mers (source proteins: CDH19, KIF1A, RAPGEF2) (Figure 7D). Among the shared proteins, we identified the Schwann cell precursor marker CDH19, and among the twenty highest expressed neural source proteins found in DFT1 + IFN‐γ but not in DFT2, we identified the Schwann cell markers myelin‐associated glycoprotein (MAG) and myelin protein zero (MPZ). Among those unique to DFT2, we found the early glial markers nerve growth factor receptor (NGFR).

## Discussion

This is the first study to identify endogenous peptides from the MHC‐I molecules of a transmissible cancer and its host. The analysis joins only a limited number of studies on MHC‐I‐derived peptides from wild species.^14^, ^16^ Here, we have defined the antigenic landscape of representative tumour cell lines, generating data to contribute to an evidence‐based approach for vaccine design. We show that DFT1 + IFN‐γ and DFT2 cells have a more limited repertoire of MHC‐I molecules compared with host cells and the peptides isolated from the tumour and host cell lines reveal a common consensus motif with a preference for leucine (L) at p3 and pΩ. The shared motif between the cell lines may be explained by a dominantly expressed MHC‐I allotype present on DFT1, DFT2 and host fibroblast cells. Alternatively, different devil MHC‐I allotypes may share a preferred peptide motif. Either possibility would contribute to the apparent tolerance of the devil immune system to these contagious cancers as the restricted MHC‐I repertoire and dominant peptide motif reduces the antigenic variation on DFT1 + IFN‐γ and DFT2 compared with host.

The presence of a hydrophobic anchor residue at pΩ is consistent with the peptide motif of other mammalian and non‐mammalian MHC‐I alleles.^11^, ^47^, ^48^ Less common among other species is the presence of an anchor residue at p3. Notable exceptions are HLA‐A*01:01, with an acidic E/D at p3^30^ and HLA‐C*04:01, with a preference for D at p3 and a secondary anchor at p2.^11^, ^31^ Our analysis of >16 000 MHC‐I alleles and homology modelling of the devil MHC‐I allele SahaI*27 against the recently described bat MHC‐I structure (Ptal‐N*01‐01)^14^ show a high level of conservation within the F pocket that is absent in the B pocket and may explain the lack of a p2 anchor in the devil peptides. Interestingly, analysis of the devil MHC‐I alleles predicts a B pocket preference for only SahaI*27 and SahaI*74/88, but a F pocket prediction for a hydrophobic residue is largely consistent across the alleles. It is possible that certain devil MHC‐I molecules bind peptides through the B pocket and utilize a p2 anchor or a p1 anchor. Given our analysis used a pan‐specific anti‐β_2_m antibody, we may have captured multiple peptide motifs, some of which are partially ‘hidden’ in our data set due to differences in expression level among MHC‐I allotypes. Using the data generated here, it should be possible to determine the exact binding properties of particular MHC‐I allotypes using structural analyses.

The use of the anti‐β_2_m antibody for immunoaffinity purification of MHC‐I molecules presents a problem for assigning specific peptide motifs to individual devil MHC‐I allotypes. At a minimum, we predict that fibroblasts have six allotypes at the cell surface and DFT1 + IFN‐γ and DFT2 have 3 and 5 functional allotypes, respectively. These alleles correlate well with our previous definition of the MHC‐I transcripts expressed by DFT1 + IFN‐γ and DFT2 and fibroblasts.^21^ However, there is no evidence of SahaI*27/27‐1 or SahaI*46 in the alpha chain fraction from DFT1 + IFN‐γ cells, despite the presence of mRNAs for these alleles in DFT1 + IFN‐γ. In addition, only trace amounts of the non‐classical MHC‐I, SahaI*UK, are present in all three cell lines, which is surprising given we have previously found mRNA transcripts in the tumour cell lines. This could be because SahaI*UK does not bind peptides, as is the case for other non‐classical MHC‐I molecules^49^, ^50^ and, without its ligand is unstable and remains sequestered in the ER.

Mammalian MHC‐I molecules generally bind to peptides, which are 8–10 amino acids in length, although several examples of different length distribution preferences have been described.^51^, ^52^ Typically, 9mers are the most abundant peptides for the majority of HLA allotypes.^12^ We found that MHC‐I molecules on DFT cells and fibroblast cells bind high numbers of both 8mers and 9mers, with DFT2 and fibroblasts having a preference for 8mers. The preference for 8mers may reflect specific binding properties of the devil MHC‐I molecules, and the preference for 9mers in DFT1 + IFN‐γ cells may be due to stimulation of these cells with IFN‐γ, which is known to change the repertoire of peptides presented when the immunoproteasome is triggered.^33^, ^53^, ^54^

MHC^+^‐DFT1 cells have become the focus of research to determine whether they are allogenic to host devils and have potential as a whole‐cell vaccine against DFT1.^18^, ^20^ Preliminary results have shown that MHC^+^‐DFT1 cells can stimulate a protective immune response in host devils, but the mechanisms are not understood, the results are variable among host devils^18^, ^20^ and a lack of known antigens has been noted.^55^ In this study, we have not identified any presented peptides with mutations specific to DFT1 or DFT2. However, we have identified four unique 8/9mer peptide sequences deriving from source proteins, which are present on the surface of both DFT1 + IFN‐γ and DFT2, but not fibroblast cells. These peptides derive from source proteins that are specific to or overexpressed in cells of the nervous system (including Schwann cell development), which supports previous studies proposing a common cellular origin for the tumours^46^ and indicates that tumour‐associated antigens may exist in both DFT1 and DFT2. However, further analysis is needed to characterize the expression of these proteins in more tissues in the Tasmanian devil and, as none of these peptides share the common motif identified (i.e. L at p3 and L at pΩ), to determine MHC‐I alleles that bind through refolding assays.

Here, we have demonstrated a restricted MHC‐I allele repertoire on transmissible cancer cells combined with a peptide binding motif similar to that found in a host cell line. The restriction of devils to an island population, a limited repertoire of MHC‐I allotypes and similarity of peptide motifs across these allotypes, is consistent with an inability to distinguish self from non‐self, thus leading to repeated emergence of transmissible cancer. However, loss of MHC‐I molecules by DFT1 suggests that there is a limit to the ability of these tumours to transmit while still expressing MHC‐I molecules, requiring further investigation.

## Conflict of interest

The authors have no competing interests.

## Supporting information


**Figure S1**. Analysis of the expression of MHC class I (MHC‐I) molecules in a devil fibroblast cell line and in devil facial tumour disease (DFT1 & DFT2) cell lines.

**Figure S2**. Peptidomics experiments on a smaller number of devil facial tumour 2 (DFT2) cells confirms dominance of 8mer peptide sequences.

## Data Availability

The peptidome and proteome data sets have been deposited to the ProteomeXchange Consortium via the PRIDE partner repository with the data set identifiers PXD020614 (peptidome) and PXD021784 (proteome). Data files for all analyses used to generate Figures 2–7 are openly available from the University of Southampton repository at https://doi.org/10.5258/SOTON/D1585.
