# Supplementary material for: The immunopeptidomes of two transmissible cancers and their host have a common, dominant peptide motif
Source: Immunology. 2021 Feb 4;163(2):169–84. doi: 10.1111/imm.13307 (PMC8114214; doi:10.1111/imm.13307)
Supplement: Supplementary file 1 — Figure S1. Analysis of the expression of MHC class I (MHC‐I) molecules in a devil fibroblast cell line and in devil facial tumour disease (DFT1 & DFT2) cell lines. [file IMM-163-169-s002.ppt]

## Slide 1
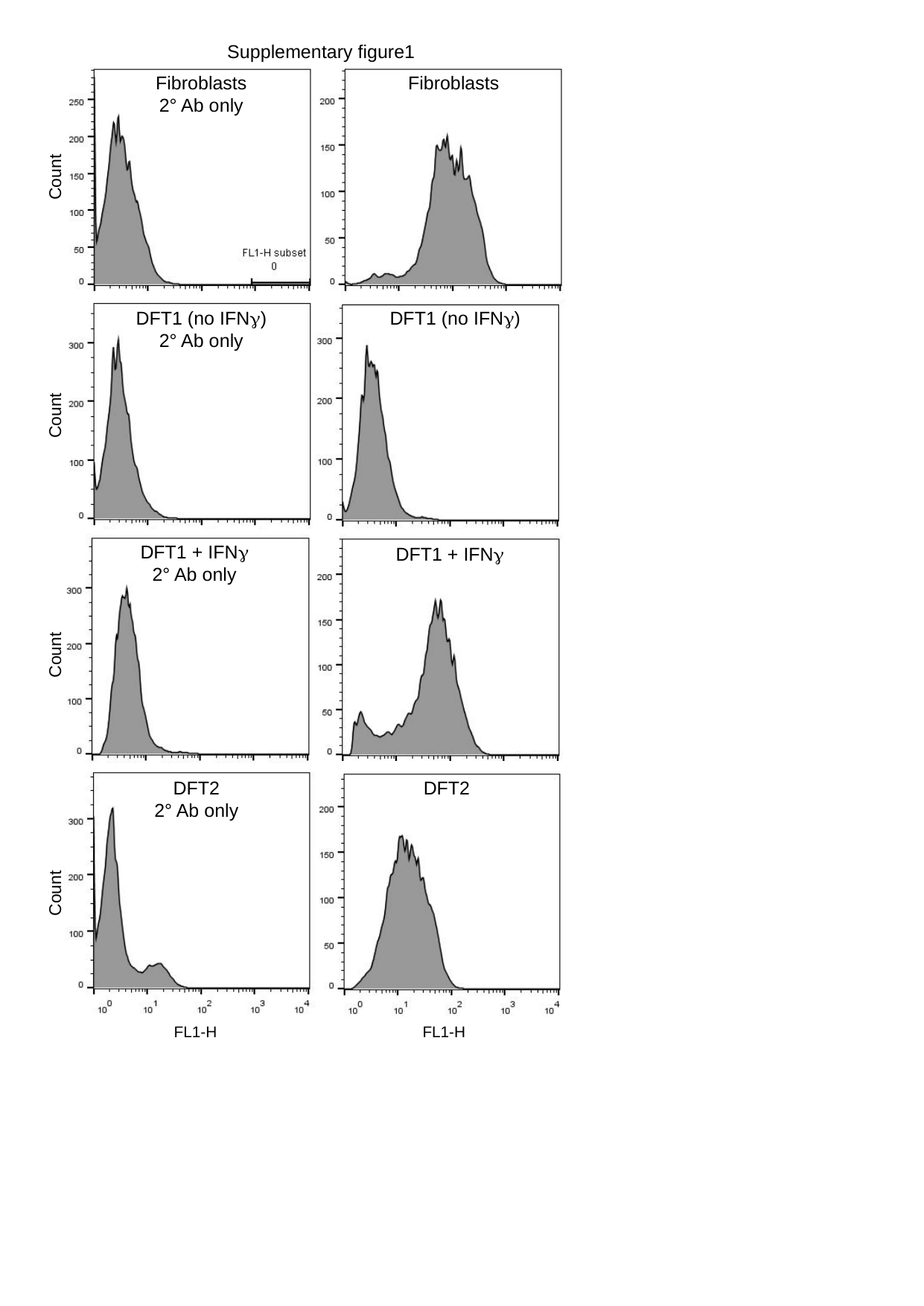

Supplementary figure1
Fibroblasts
2° Ab only
Fibroblasts
Count
DFT1 (no IFNγ)
DFT1 (no IFNγ)
2° Ab only
Count
DFT1 + IFNγ
2° Ab only
DFT1 + IFNγ
Count
DFT2
2° Ab only
DFT2
Count
FL1-H
FL1-H
