# Supplementary figures and images for: The immunopeptidomes of two transmissible cancers and their host have a common, dominant peptide motif
Source: Immunology. 2021 Feb 4;163(2):169–84. doi: 10.1111/imm.13307 (PMC8114214; doi:10.1111/imm.13307)

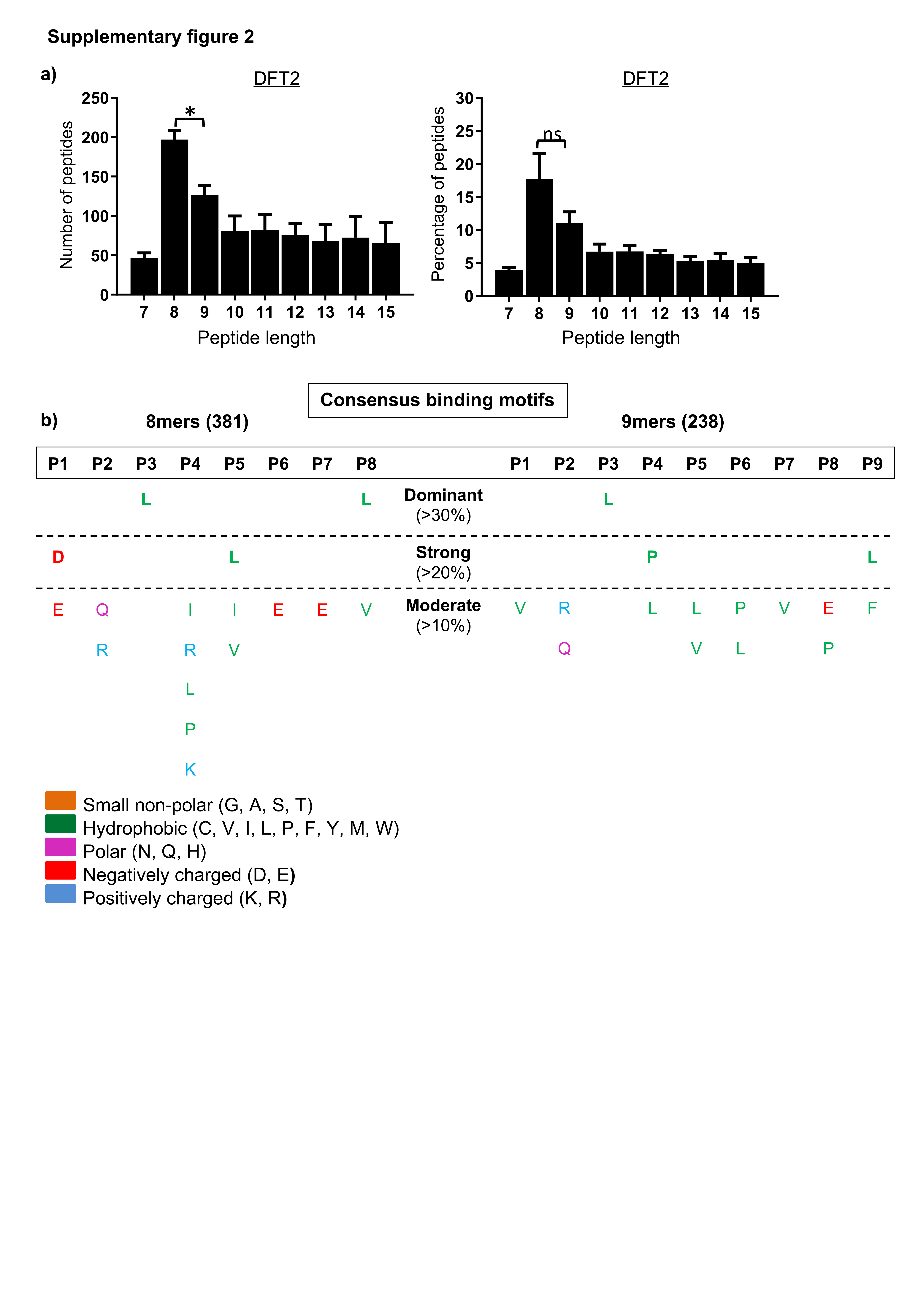

Supplement: Supplementary file 2 — Figure S2. Peptidomics experiments on a smaller number of devil facial tumour 2 (DFT2) cells confirms dominance of 8mer peptide sequences. [file IMM-163-169-s001.tif]
